# Evidence for decreased maladaptive guilt following PCIT-ED for depression as measured by story stem narratives: a promising method for preschool self-disclosure of emotions and experiences of parenting

**DOI:** 10.1007/s00787-026-02996-7

**Published:** 2026-03-18

**Authors:** Judson Ellis, Joan Luby, Annika Agrawal, Anna Freeman, Kirsten Gilbert, Diana Whalen, Rebecca Tillman, Meghan Rose Donohue

**Affiliations:** Department of Psychiatry, Washington University School of Medicine, Saint Louis, USA

**Keywords:** Preschool, Guilt, Depression, Parenting Styles, Narratives

## Abstract

Preschool-Onset Major Depressive Disorder (PO-MDD) has been validated in children as young as 3 years old. Guilt is a highly specific symptom of the disorder, and maladaptive parenting behaviors are a key risk factor. The purpose of this study is to investigate the utility of the MacArthur Story Stem Battery (MSSB), a narrative technique, as a valid tool through which depressed preschoolers may express their own internal representations of their maladaptive guilt and their caregiver’s parenting. MSSB narratives were coded for children’s representations of their own guilt and their caregivers’ parenting styles before and after an evidence-based dyadic therapy for PO-MDD—Parent-Child Interaction therapy-Emotional Development (PCIT-ED)—and compared to Waitlist controls. Results indicate that narrative guilt themes significantly decreased in children assigned to PCIT-ED compared to a Waitlist control group, and that parenting style themes correlated significantly with observed parenting behavior. These findings validate the use of MSSB narrative techniques to track changes in preschoolers’ maladaptive guilt following treatment for depression. Findings also provide validation of this method to capture preschool children’s experiences of parenting.

**Supplementary Information:**

The online version contains supplementary material available at 10.1007/s00787-026-02996-7.

## Introduction

Depression is known to arise as early as the preschool period [27], with maladaptive guilt as a central symptom and parenting behavior as a key risk factor [10, 30]. Determining a valid method to systematically assess the child’s internal feeling states and perceptions of parenting is critical to accurately diagnose and monitor a child’s symptoms of and risk for Preschool-Onset Major Depressive Disorder (PO-MDD). Structured narrative techniques such as the MacArthur Story Stem Battery (MSSB; [7]) hold promise as they are open-ended and do not require children to have direct insight into their feelings or perceptions. The purpose of this study is to investigate the utility of the MSSB as a valid tool through which depressed preschoolers can disclose representations of their own maladaptive guilt and of their caregivers’ parenting, and to explore whether these representations change following a dyadic psychotherapuetic treatment for PO-MDD.

### Major depressive disorder in preschool children

PO-MDD has been validated in children as young as 3 years old [18]. It affects 1% to 2% of children ages 3 to 5 [8, 17, 46] and is characterized by a specific and stable symptom constellation [27]. Maladaptive guilt has emerged as a sensitive and highly specific symptom of PO-MDD [30]. As an emotional response to transgression which stimulates empathy and a sense of personal responsibility, guilt typically functions adaptively, especially when it is proportionate to the transgression and motivates reparative prosocial behaviors [40]. However, maladaptive guilt arises when these feelings are excessive, inappropriate to the situation, or do not result in adaptive resolution of the problem but linger and exacerbate distress [41]. PO-MDD, though a heritable disorder, is also influenced by environmental factors such as the child’s experiences of their caregivers’ parenting [13], with elevated parental criticism and negativity established as risk factors [10]. Specifically, children who experience authoritarian parenting styles, defined by harshness and strict rule-setting, are more likely to develop symptoms of depression compared to those who experience authoritative parenting, defined by warmth and flexible but clear boundaries [24], or permissive parenting, defined by warmth and flexibility without clear boundaries [39]. Given that early depressive symptoms predict later negative outcomes [6], it is critical to refine our ability to diagnose key symptoms of depression and identify known risk factors such as parenting at the earliest possible developmental stage.

### Young children’s self-report of their own psychiatric symptoms

The diagnosis of psychopathology in preschoolers has long relied almost exclusively on parent and teacher report, as children under 6 years old have been widely considered developmentally unable to serve as valid reporters of their own mental states [34]. However, adult caregivers are limited in their ability to detect highly internal symptoms of depression such as guilt, worry, and anhedonia [11], leading them to underreport these symptoms compared to older children [38]. Caregivers are also known to underreport their own negative parenting behaviors, with a meta-analysis by Korelitz and Garber [26] finding that parents tended to rate their parenting behaviors more positively than either child-report or observational measurements. Consistent with this, caregivers’ reports of their own parenting are poor predictors of child outcomes compared to observational measures [48] or child-report [19].

In light of these discrepancies, several age-adjusted interview techniques have been developed to attempt to accurately elicit self-report in children aged 6 and under. Because preschoolers are susceptible to the suggestive nature of specific questions [22], the Berkeley Puppet Interview (BPI) was designed to obtain child self-report without forced choice questions. In the BPI, two puppets present opposing choices (e.g. “I’m a happy child”/“I’m not a happy child”) and the child is asked an open-ended question about which feeling state best matches their own. Children’s coded responses from the BPI correlate with parent-reported depression severity in children as young as four years old [1]. However, Luby et al. [29] demonstrated that while puppet interview responses correlate well with basic symptoms of depression, such as crying, they may not validly tap more complex symptoms central to the diagnosis of PO-MDD such as maladaptive guilt. Alternative interview methods utilizing cartoons are similarly limited. The Violence Exposure Scale (VEX-R) uses cartoons to gauge a young child’s experience with violence [37], but it has not been validated for the measurement of internal feeling states. Overall, age-adjusted interview methods are still limited by preschoolers’ inability to identify and label their more complicated internal feeling states [36]. For this reason, non-interview methods of self-disclosure have been developed. The Family Drawing Paradigm (FDP) asks a child to draw their family and identify the attachments within their drawing [20]. However, it is limited by developing fine motor skills in young children [35]. These limitations underscore the need for a developmentally appropriate method of self-disclosure of preschoolers’ complex internal emotional states.

### Self-disclosure through narratives: the MSSB

The MacArthur Story Stem Battery (MSSB) is a well-validated narrative tool for children aged 3 to 7 years that provides “information about children’s views of themselves and the world” [42]. In the MSSB, experimenters provide standardized ‘story stems’ (i.e., beginnings) which set up the themes of a story narrative using play figures and furniture, before asking children to finish the stories. Through narratives, children unconsciously reveal how they expect certain scenarios will evolve based on their internal representations (or inner expectations) of themselves and others [16]. This method prompts the displacement of the child’s complex feelings like maladaptive guilt onto the characters without having to identify or label them. Responses are coded from videotapes, allowing a systematic analysis of young children’s internal states. MSSB story stems can be adapted to tap specific constructs of interest. Our study examines stories involving child transgressions, designed to elicit the child’s maladaptive guilt and reparative responses (including prosocial attempts to mend their transgressions, alleviating guilt; [12]), as well as parenting responses to the child’s transgression (e.g., punitive vs. comforting).

Research supports the validity of MSSB narrative themes as indicators of child psychopathology. For example, children with more parent-reported externalizing symptoms were more likely to express themes of aggression and injury in their narratives [42], and one study of 5-year-olds found that children’s negative expectations of themselves, others, and experiences coded from their narratives significantly predicted parent- and teacher-reports of depression and anxiety symptoms [43]. Additionally, a study by Luby et al. [30] found significant associations between narrative guilt and shame themes, and between shame themes and depression severity, suggesting that the MSSB may be a valid tool through which to assess children’s self-conscious emotions. To date, no studies have investigated changes in narrative guilt themes following treatment for PO-MDD, despite maladaptive guilt being a highly specific symptom of the disorder.

The literature also supports associations between preschool depression and parenting themes expressed in MSSB narratives. In one study, preschoolers with depression expressed more negative and disciplinarian maternal representations than children without depression [5]. These negative internal representations were correlated with later observational measurements of maternal non-supportive parenting behaviors and negative affect, supporting the validity of children’s narrative representations of parenting. No study to date has examined whether children’s parenting representations reflect specific parenting styles, or whether these representations might change following interventions that directly target parenting. In sum, the MSSB may provide a means of more accurately measuring preschool children’s guilt and parenting experiences, which would be critical to our ability to identify symptoms of and risk factors for PO-MDD.

### PCIT-ED treatment for PO-MDD

Randomized controlled trial evidence has demonstrated the efficacy of Parent-Child Interaction Therapy with an Emotional Development module (PCIT-ED) in treating PO-MDD, as preschoolers randomized to the PCIT-ED group evidenced lower rates of depression, less severe depression, and less impairment compared to children randomized to a Waitlist control condition [31]. PCIT-ED targets the caregiver-child dyad, improving emotional regulation and reducing maladaptive guilt. Standard PCIT includes two main components, Child-Directed Interaction (CDI), which coaches caregivers in following the child’s lead during interactions with their child, and Parent-Directed Interaction (PDI), which guides caregivers in the use of clear, direct commands and in managing challenging child behaviors. These modules improve caregiver-child relationship quality and encourage authoritative parenting styles. The Emotional Development (ED) module, novel to PCIT-ED, enhances the child’s ability to identify and understand their emotions. This is achieved in part by teaching caregivers to tolerate and validate the child’s negative emotions and to serve as an external emotion regulator and coach. Crucially, the novel ED module may be uniquely effective at reducing child depression, as remission-associated changes in child neural response to reward and parental response to child emotional expression have been found to be specifically attributable to the ED module [33]. Overall, the efficacy of PCIT-ED underscores that the caregiver-child dyad is a key agent of change for treating PO-MDD.

The MSSB provides a tool for capturing features of the caregiver-child dyad from the child’s perspective. One study of MSSB narratives demonstrated that children who expressed more negative representations of their mothers prior to PCIT-ED were less likely to remit from depression following treatment [15], highlighting that children’s internal representations may be moderators of treatment response. However, no study has examined how these internal representations might change with administration of PCIT-ED. Furthermore, this study relied on a non-specific coding scheme that was limited only to children’s positive and negative parental representations. For the current study, we designed a detailed coding scheme that captures representations of specific parenting styles, allowing us to more deeply explore children’s representations of parenting styles and examine potential treatment effects.

### Overview of the current study

In our study, MSSB narratives were administered to depressed preschoolers before and after PCIT-ED treatment. Story stems chosen for this study were designed to elicit two key internal representations directly targeted by the treatment: children’s maladaptive guilt and children’s experiences of their caregivers’ parenting. We had two primary goals: first, to determine whether MSSB narratives validly measure children’s maladaptive guilt and experiences of parenting styles, and second, to investigate whether these factors change following PCIT-ED treatment.

We hypothesized that maladaptive guilt narrative themes would correlate with parent-reported guilt measures and that parenting style themes would correlate with observations of parenting. Further, we hypothesized that following PCIT-ED, maladaptive guilt themes and authoritarian parenting themes would decrease, while authoritative parenting themes would increase.

## Method

### Participants

Participants were preschoolers (age 3.0–6.11) who met criteria for depression and were randomized into the PCIT-ED randomized controlled trial. Children were recruited from pediatricians’ offices, daycares, preschools, and mental health facilities in the greater Saint Louis Metropolitan Area using the Preschool Feelings Checklist (PFC; [28]) to identify children at high risk of depression (≥ 3 items endorsed). Children who subsequently met criteria for depression were invited to enroll, and were randomized to either immediate treatment (PCIT-ED group, *n* = 114) or an 18-week Waitlist (WL group, *n* = 115), after which they were offered therapy. Study exclusion criteria were autism spectrum disorder, neurological or chronic medical disorder, significant developmental delays, or current antidepressant or psychotherapy treatment. Of the 229 subjects randomized into the PCIT-ED study, all but three subjects completed the story stems of interest at the Baseline assessment.

Of these 226 subjects, 49 did not have narratives data available at the Post assessment; this included 38 subjects who did not complete the Post assessment, 3 with a parent-only phone assessment, as well as 8 who either refused to complete narratives, were mistakenly re-administered the Baseline narratives, or whose assessment was not successfully video recorded. Only subjects with narratives data available at both time points (*N* = 177) were coded for parenting styles. Once coded, individual narratives were excluded for two reasons: experimenter error (e.g., over-prompting or failing to give the required prompt; *N* = 47), complete child disengagement (i.e., the child never engages with the prompt; staying completely silent or telling an unrelated story; *N* = 15), or both (*N* = 3). Regarding analyses of parenting styles, exclusion of narratives resulted in sample sizes of *N* = 154 for analyses including only Baseline narratives data and *N* = 128 for analyses including both Baseline and Post narratives data (wherein narratives coded at either Baseline or Post could be grounds for exclusion). Regarding analyses of guilt/reparation codes (in which one additional story stem was coded at each time point and subjects were excluded from analysis), exclusion of narratives resulted in sample sizes of *N* = 149 for only Baseline and *N* = 122 for Baseline and Post (see CONSORT diagram, Online Resource 1).

### Course of treatment

Parent-Child Interaction Therapy-Emotion Development (PCIT-ED) is an empirically supported treatment for PO-MDD. PCIT-ED is an adaptation of PCIT, with the addition of a novel Emotion Development (ED) module. PCIT-ED includes 20 sessions conducted over 18 weeks. The treatment included 6 sessions of child-directed interaction (CDI), focused on positive child-led play, followed by 6 sessions of parent-directed interaction (PDI), focused on authoritative boundary setting, and 8 sessions of the ED module, focused on emotion regulation. Caregivers were coached in real time while interacting with their children through the use of a small microphone and one-way mirror, allowing a therapist to modify parental behavior in vivo [31]. Therapist training protocol, intervention fidelity monitoring procedures, and number of sessions completed are described in detail by Luby et al. [31].

### Assessments

Participants and their parents participated in an in-person Baseline assessment and again in an in-person Post assessment, which took place after completion of therapy for children randomized to PCIT-ED and approximately 18 weeks after Baseline for children on the Waitlist. The measures described below are the subset of the measures administered at these assessments that are included in the current analyses.

### Measures

#### Children’s depressive symptoms

The Kiddie Schedule for Affective Disorders and Schizophrenia – Early Childhood (KSADS-EC; [21]), a semi-structured clinical interview, was administered to the caregiver to assess the child’s DSM-5 symptoms and diagnoses. The current study examined depression severity, which was calculated as the number of 9 core symptoms of MDD endorsed. Questions probed the presence of MDD over the past month, but to meet criteria for MDD, symptoms had to have occurred over the past 2 weeks. The maladaptive (i.e., excessive and/or inappropriate) guilt symptom in the MDD supplement of the KSADS-EC was also examined and dichotomized for analyses as absent vs. present (i.e., subthreshold or threshold). The KSADS-EC was videotaped and reviewed for rater drift, and satisfactory inter-rater reliability was established (k = 0.74).

#### Narrative task and coding

Two separate MSSB narratives involving transgression by the child were collected at both Baseline (Band-Aid and Spilled Juice) and Post (Hot Soup and Cookie Jar). See Online Resource 2 for detailed descriptions. Child participants were asked to finish each story, enabling them to express the child and caregiver’s emotional and behavioral responses to transgression.

The guilt/reparation code from the MSSB coding scheme captured any instance of guilt or reparative behavior from any character within the narrative. This was a dichotomous code, with 0 or 1 representing the absence or presence of guilt or reparation. The final variable measured in this analysis was a dichotomous score of whether guilt/reparation was present in either transgression narrative at each given time point.

The guilt/reparation code used in the original MSSB coding scheme aggregated instances of child guilt affect with reparative behaviors. Since we were also interested in examining guilt affect separate from reparative behavior, we created an updated coding manual which separated guilt affect, adaptive reparation (reparative behaviors commensurate with the transgression), and maladaptive reparation (behaviors attempting to repair transgression which involved excessive perseveration or disproportionate response to the transgression) into unique codes described in Online Resource 3. We used this coding scheme to analyze the Band-Aid (at Baseline) and Hot Soup (at Post) story stems. Maladaptive reparation cell sizes were too small to be analyzed further (*n* = 4). Guilt affect and adaptive reparation codes were examined in Online Resource 3, to examine whether any significant findings were driven most by affect versus behavior.

Given that parenting codes in the MSSB scheme are limited to very broad positive and negative representations of caregivers in narratives, we designed a more detailed coding scheme to deeply explore parenting styles in the narratives, given the literature linking authoritative and authoritarian parenting styles to lower and higher levels of childhood depression, respectively. We used this coding scheme to analyze the Band-Aid (at Baseline) and Hot Soup (at Post) story stems, as these were the most directly comparable with respect to parenting behaviors. Parenting style codes were conceptualized in accordance with Baumrind’s framework [4]. *Authoritative Parenting* was coded in response to firm parenting and/or clear expectation setting that simultaneously displayed warmth and flexibility. *Authoritarian Parenting* was coded in response to firm parenting with the absence of warmth or the presence of excessive harshness and strictness. *Permissive Parenting* was coded in response to the presence of warmth but the absence of clear expectations and/or firm parenting behaviors. *Uninvolved Parenting* was coded for when caregivers in the narrative displayed no interest in the scene or failed to appear in the child’s narrative entirely. Each parenting style was coded dichotomously as 0 (absent) or 1 (present). Because *Uninvolved Parenting* was present in only 5 narratives, this variable was excluded from analysis.

Coders were blind to children’s treatment group and study hypotheses. After a training period, during which a small group of coders (5 in the original MSSB coding process, 4 in the more detailed parenting styles codes) reviewed the manual, discussed interpretation of specific codes, and observed and practiced example codes with a master coder until 75% reliability was achieved, after which coders independently rated each videotaped narrative. During both coding processes, one master rater rated 25% of random videos to prevent rater drift and ensure that inter-observer agreement was maintained over time. Final inter-rater reliability testing demonstrated at least 75% agreement for all codes.

#### Observations of parenting

The child and caregiver completed two parent-child interaction (PCI) tasks designed by Kochanska and Aksan [25] at both Baseline and Post, although only the Baseline PCI tasks of Marble Run and Etch-A-Sketch were included in this analysis. The Marble Run task required the parent-child dyad to replicate a photo of a standing marble run by building it under a time limit. In the Etch-A-Sketch task, the caregiver and child each controlled a dial and worked together through a maze. The design of both tasks induced mildly stressful situations and negative emotions, requiring the caregiver to assist the child. Each task had an approximate time-limit of 3.5 min.

The coding system was an adapted version of the Dyadic Parent-Child Interactions in Early Childhood, PCIT-ED edition manual [32, 44]. The system independently coded parental behavior and affect. Four variables were examined in this study: *Duration of Negative Parenting Behavior*, *Duration of Negative Parenting Affect*, *Duration of Positive Parenting Behavior*, and *Duration of Positive Affect Composite*. Each variable was a mean duration of parent expressions (duration of each code divided by total length of combined tasks) of the individual behavior or affect codes. For example, the *Duration of Negative Parenting Behavior* included the behavior codes of Negative Physical Discipline, Negative Verbal Discipline, Intrusiveness, and Disengagement. See Online Resource 4 for further distinctions.

Coders were blind to study hypotheses and treatment group. After a training process during which a total of 25 coders reviewed and discussed codes, practiced coding with example videos, and achieved greater than 80% reliability with the two master coders, coders independently rated videos. Master coders coded 20% of random videos to verify inter-rater reliability was maintained over time.

#### Maladaptive guilt and reparation

The My Child is a 100-item parent-report questionnaire that measures children’s self-conscious emotions. Each item is rated on a 1–7 Likert scale, with 1 = *extremely untrue of child* and 7 = *extremely true of child*. Items are collapsed into 10 subscales by taking the mean of item scores. For the analyses presented here, Guilt Feelings was calculated as the sum of 4 subscales: Symbolic Reproduction, Guilt, Concerned by Others’ Transgressions, and Sensitive to Themes, and Guilt Reparation was calculated as the sum of 6 subscales: Amends, Internalizing Conduct, Confession, Empathy, Concern, and Apology [30]. Notably, higher Guilt Feelings scores have been found to function maladaptively within samples of depressed preschool children [14, 30].

#### Income-to-needs ratio

An income-to-needs ratio was calculated as the total family income divided by the federal poverty level based on family size in the year of data collection. An income-to-needs ratio of < 1 indicates living in poverty.

#### Child behavior

The Child Behavior Checklist (CBCL [2]) is a caregiver-report measure of dimensional psychopathology severity in children. From the CBCL, we can calculate scores of externalizing and internalizing pathology in children, as well as scores of inattention.

#### Child IQ

The Kaufman Brief Intelligence Test Second Edition (KBIT-II [23]) is a brief screen measuring children’s verbal and nonverbal intelligence and providing a composite IQ score.

### Analytic Plan

Preliminary analyses were conducted to determine whether there were significant demographic differences between the participants included and excluded from our analyses. We examined whether participants who completed Post narratives differed from participants who did not complete Post narratives in their baseline demographic or psychopathologic characteristics, using t-test for continuous variables and Chi-square tests for categorical variables. Then, to examine why certain children did not engage with the content of the narratives, we investigated whether participants with complete disengagement during narrative tasks differed in KBIT-II IQ score or CBCL inattention T-scores compared to those without complete disengagement using t-tests.

Analyses of study hypotheses first examined whether parent-reported depression and guilt scores at baseline were associated with guilt/reparation on the baseline narratives. Linear regression was used to examine whether narrative guilt themes were associated with parent-reported depression severity on the KSADS-EC and guilt feelings and guilt reparation on the My Child questionnaire, and logistic regression was used to examine whether narrative guilt themes were associated with meeting criteria for maladaptive guilt on the KSADS-EC. Next, linear regression models were used to examine whether parenting style narrative themes were associated with baseline PCI composite codes. All models covaried for baseline age and sex at birth. Multiple comparisons were accounted for using the False Discovery Rate (FDR) correction.

Then, we investigated whether guilt and parenting narrative themes differed at Post as a function of randomization group (PCIT-ED vs. Waitlist). A logistic regression model was conducted with narrative guilt themes at Post as the outcome and randomization group as the independent variable. We then ran supplemental logistic regression analyses to examine whether any significant result was most driven by change in guilt affect versus reparative behavior (Online Resource 3). In addition, linear regression models examined the three parenting styles (Authoritative, Authoritarian and Permissive) as outcomes with randomization group as the independent variable. Models covaried for baseline age, sex at birth, and the theme of interest (i.e., guilt or parenting) in the baseline narratives.

## Results

Baseline characteristics of the sample included in the analyses of study hypotheses are displayed in Table 1. Participants were 5.33 (SD = 1.00) years old, 36% female, 11% Hispanic, and 79% White race.

**Table 1:** Baseline characteristics of the sample (*N* = 154)

Demographics	Total *N*	Mean	SD
Age	154	5.33	1.00
Income-to-needs ratio	153	3.12	1.28
	**Total N**	**%**	**N**
Female sex	154	35.7	55
Hispanic ethnicity	154	11.0	17
Race	154		
White		79.2	122
Black		8.4	13
Asian		0.7	1
Multiracial		11.7	18
**Narratives Codes - Child**	**Total N**	**%**	**N**
Guilt/Reparation	149	33.6	50
**Narratives Codes – Parenting Styles**	**Total N**	**%**	**N**
Authoritative parenting	154	51.3	79
Authoritarian parenting	154	7.8	12
Permissive parenting	154	8.4	13
Uninvolved parenting	154	3.3	5
**Parent-Child Interaction**	**Total N**	**Mean**	**SD**
Positive affect duration	152	0.11	0.12
Negative affect duration	152	0.06	0.08
Positive behavior duration	152	0.45	0.07
Negative behavior duration	152	0.05	0.06

Online Resource 5 compares baseline characteristics of participants with and without Post narratives data, demonstrating that subjects without Post narratives had significantly higher internalizing scores on the Child Behavior Checklist (CBCL), 70.82 (SD = 8.85) compared to 66.21 (SD = 7.33), but did not have significantly greater MDD severity by parent report.

Online Resource 6 compares the IQ and inattention scores of participants who displayed complete disengagement during any of their narratives with those who did not. Children who demonstrated complete disengagement did not differ in their inattention scores on the CBCL compared to subjects without complete disengagement; however, they had significantly lower IQ scores (96.73 [SD = 16.36] vs. 107.60 [SD = 13.58]), suggesting that they may have had more difficulty understanding the MSSB task.

Table 2 compares parent-reported child depression and guilt measures between participants whose baseline narratives contained the guilt/reparation theme versus those whose narratives did not contain this theme. Participants whose narratives included guilt/reparation had significantly higher parent-reported reparation scores on the My Child than subjects not showing guilt/reparation.

**Table 2:** Baseline parent-reported guilt measures by guilt narratives codes covarying for baseline age and sex at birth

Baseline Child Characteristics	No Baseline Narratives Guilt/Reparation (*N* = 99)		Baseline Narratives Guilt/Reparation (*N* = 50)		Baseline Narratives Guilt/Reparation vs. No Baseline Narratives Guilt/Reparation		
	Mean	SD	Mean	SD	t	*p*	FDR *p*
Depression severity	5.55	1.53	5.58	1.43	−0.55	0.5828	0.5828
My Child guilt feelings	17.92	2.69	18.54	2.33	1.72	0.0884	0.1768
My Child guilt reparation	23.53	5.07	25.88	4.88	2.59	0.0105	0.0420
	**%**	**N**	**%**	**N**	**c**^**2**^	**p**	**FDR p**
KSADS-EC guilt	50.5	50	62.0	31	1.86	0.1726	0.2301

Table 3 details associations between baseline parenting styles, narrative themes, and observational parent behavior. Participants whose narratives contained authoritative parenting themes had caregivers who were observed to display significantly longer durations of positive behavior and shorter durations of negative behavior during the PCI tasks. Participants whose narratives contained authoritarian parenting themes had caregivers who displayed significantly longer durations of negative behavior and shorter durations of positive behavior during PCI.

**Table 3:** General linear models of baseline parent-child interaction composite scores by baseline narratives parenting styles codes covarying for baseline age and sex at birth (*N* = 152)

Parent Codes – Styles	DV = PCI Positive Affect Duration				
	Estimate	SE	t	*p*	FDR *p*
Authoritative parenting	0.038	0.021	1.79	0.0757	0.2282
Authoritarian parenting	0.040	0.037	1.09	0.2774	0.3699
Permissive parenting	0.029	0.036	0.82	0.4154	0.4154
	**DV = PCI Negative Affect Duration**				
**Parent Codes – Styles**	**Estimate**	**SE**	**t**	**p**	**FDR p**
Authoritative parenting	−0.011	0.013	−0.85	0.3979	0.7465
Authoritarian parenting	0.029	0.023	1.27	0.2050	0.7465
Permissive parenting	−0.007	0.022	−0.32	0.7465	0.7465
	**DV = PCI Positive Behavior Duration**				
**Parent Codes – Styles**	**Estimate**	**SE**	**t**	**p**	**FDR p**
Authoritative parenting	0.039	0.012	2.90	0.0042	0.0084
Authoritarian parenting	−0.063	0.021	−3.05	0.0027	0.0084
Permissive parenting	−0.006	0.021	−0.31	0.7585	0.7585
	**DV = PCI Negative Behavior Duration**				
**Parent Codes – Styles**	**Estimate**	**SE**	**t**	**p**	**FDR p**
Authoritative parenting	−0.028	0.010	−2.70	0.0077	0.0154
Authoritarian parenting	0.051	0.018	2.87	0.0046	0.0154
Permissive parenting	0.001	0.018	0.07	0.9421	0.9421

**Fig. 1:**
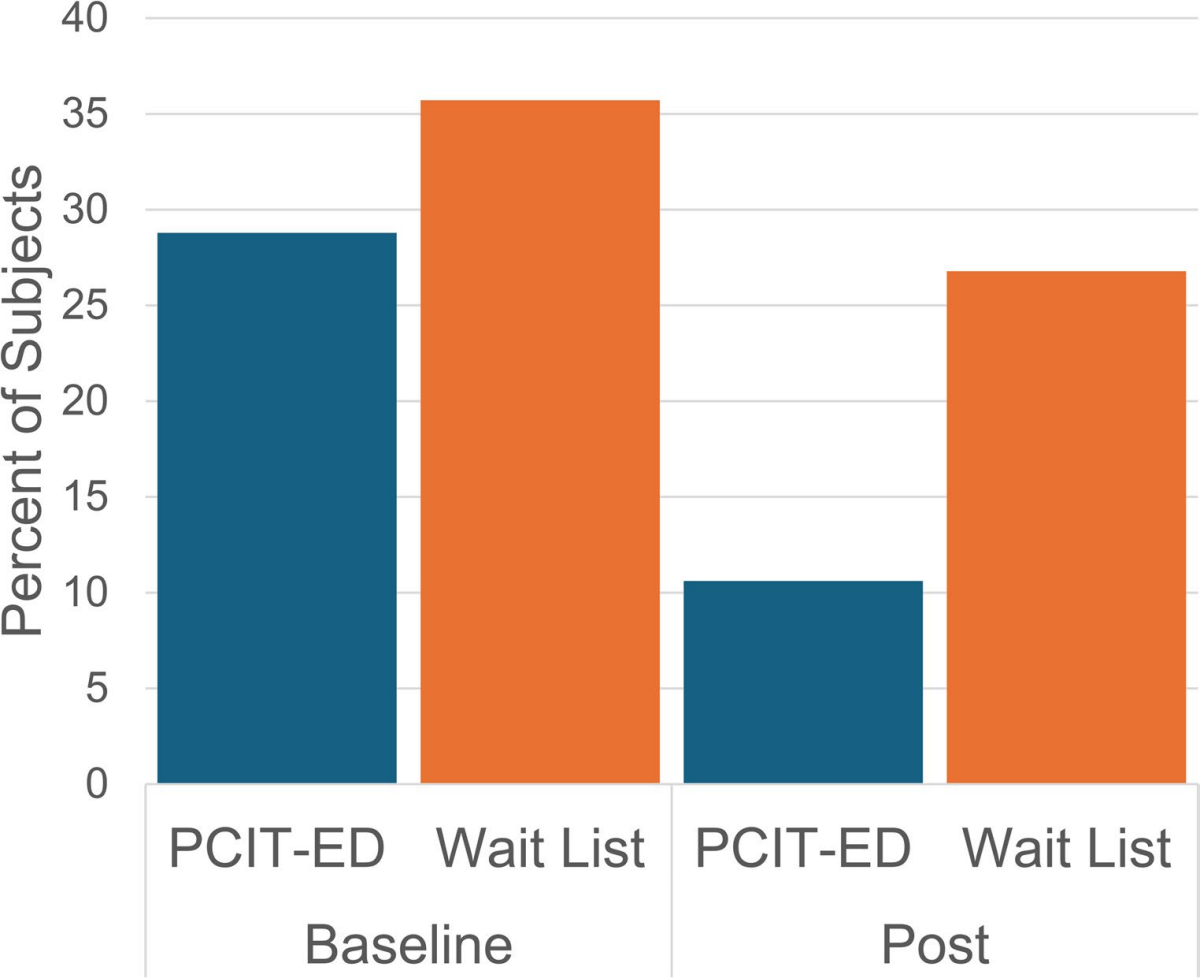
Change in guilt/reparation from baseline to post by randomization group

As shown in Table 4, participants randomized to WL were 3.15 times more likely to exhibit guilt/reparation themes in their narratives at the Post timepoint compared to participants randomized to PCIT-ED, even when accounting for the presence of the guilt/reparation theme in the baseline narratives. Figure 1 provides a visualization of this difference. Supplemental analyses examining guilt affect and reparation separately were not significant (Online Resource 3). Parenting style narrative themes at Post were not found to significantly vary by randomization group (Table 5).

**Table 4:** Logistic regression model of guilt/reparation in the post narratives by randomization group covarying for baseline age, sex at birth, and guilt in the baseline guilt narratives (*N* = 122)

	Estimate	SE	OR (95% CI)	c^2^	*p*
Intercept	−0.543	1.373	--	0.16	0.6927
Baseline age	−0.165	0.253	0.85 (0.52, 1.39)	0.43	0.5144
Female sex	0.115	0.255	1.26 (0.46, 3.42)	0.20	0.6527
Baseline guilt/reparation	0.484	0.254	2.63 (0.97, 7.13)	3.62	0.0572
Waitlist vs. PCIT-ED	0.573	0.258	3.15 (1.14, 8.66)	4.91	0.0266

**Table 5:** Linear regression of parenting styles and parent affects in the narrative guilt stories by randomization group (PCIT-ED vs. Waitlist) (*N*=128)

DV = Post Authoritative Parenting	Estimate	SE	OR (95% CI)	c^2^	*p*
Intercept	−1.595	1.119	--	2.03	0.1539
Baseline age	0.189	0.207	1.21 (0.81, 1.81)	0.84	0.3601
Female sex	0.254	0.209	1.66 (0.73, 3.77)	1.48	0.2236
Baseline authoritative parenting	0.172	0.205	1.41 (0.63, 3.15)	0.71	0.4003
Waitlist vs. PCIT-ED	0.162	0.194	1.38 (0.65, 2.96)	0.70	0.4029
**DV = Post Authoritarian Parenting**	**Estimate**	**SE**	**OR (95% CI)**	**c**^**2**^	**p**
Intercept	−3.153	1.778	--	3.15	0.0761
Baseline age	0.117	0.294	1.13 (0.63, 2.00)	0.16	0.6897
Female sex	−1.310	0.700	0.07 (0.01, 1.13)	3.50	0.0615
Baseline authoritarian parenting	0.671	0.401	3.83 (0.80, 18.42)	2.81	0.0939
Waitlist vs. PCIT-ED	0.154	0.311	1.36 (0.40, 4.60)	0.24	0.6218
**DV = Post Permissive Parenting**	**Estimate**	**SE**	**OR (95% CI)**	**c**^**2**^	**p**
Intercept	−3.290	1.401	--	5.51	0.0189
Baseline age	0.375	0.249	1.46 (0.89, 2.37)	2.28	0.1310
Female sex	0.360	0.231	2.05 (0.83, 5.08)	2.42	0.1200
Baseline permissive parenting	0.042	0.422	1.09 (0.21, 5.68)	0.01	0.9214
Waitlist vs. PCIT-ED	0.031	0.230	1.07 (0.43, 2.63)	0.02	0.8918

## Discussion

The present study investigated the role of MSSB narratives in capturing depressed preschoolers’ internal representations of guilt and parenting styles and examined whether these representations change following PCIT-ED treatment for PO-MDD. In line with study hypotheses, guilt narrative themes measured by the MSSB were significantly reduced in subjects who completed PCIT-ED compared to those on the Waitlist. Further, parenting style themes measured by the MSSB at baseline (prior to randomization) were significantly concurrently associated with measures of observed parenting. Overall, these findings suggest that PCIT-ED reduces depressed children’s maladaptive guilt, and that the MSSB may be a useful method of self-disclosure of self-conscious emotions and experiences of being parented in preschool children.

The presence of guilt themes at baseline was significantly concurrently associated with greater caregiver-reported child reparative behaviors, but was not significantly associated with caregiver-reported child guilt feelings, despite a trend in the expected direction. The former result was expected, as the narrative code used in our study encompassed both guilt affect and reparative behaviors. While reparative behaviors often operate adaptively, they may also operate maladaptively in depressed children, becoming excessive and perseverative, and failing to prosocially resolve the transgression [47]. The positive, but non-significant association between the guilt/reparation code and caregiver-reported guilt feelings provides support that our primary guilt theme indexes maladaptive guilt in our study, as several studies indicate that caregiver-reported guilt feelings reflect a maladaptive form of guilt [14, 30]. Moreover, there was a trend in our data in which a greater proportion of children whose narratives contained guilt/reparation themes met diagnostic criteria for maladaptive guilt, a specific depressive symptom measured by caregiver-report, than children whose narratives did not contain this theme. Still, in light of the well-known limitations of caregiver report in capturing the child’s more subtle, internal experience of guilt, especially in the absence of overt reparative behaviors [11], future studies should compare depressed preschoolers’ guilt and reparation themes with behavioral observations of children’s guilt and reparative behaviors following in vivo transgressions, which may be more comprehensive measures than caregiver report.

Crucially, our study found that children’s narrative guilt themes, encompassing guilt feelings and reparative behaviors, decreased following PCIT-ED. Supplemental analyses examining guilt affect and reparation themes separately yielded no significant results, suggesting that no one individual facet of the guilt code drove these findings. Furthermore, multiple trends of the guilt code indicate that it reflects maladaptive guilt. Prior to this study, very little work had examined whether treatments for depression specifically reduced maladaptive guilt, although there is evidence in adults that effective depression treatments may be mediated by reductions in guilt feelings [3]. PCIT-ED directly targets maladaptive guilt through modules that elicit children’s guilt feelings through in vivo interactions and coach children and caregivers to respond adaptively to these feelings. Our study is the first to demonstrate that PCIT-ED treatment for PO-MDD can reduce children’s maladaptive guilt. If replicated, these findings could further validate the efficacy of PCIT-ED, and support the use of narrative tools to obtain preschool self-disclosure in a clinical setting, aiding in the identification and monitoring of internal symptoms of PO-MDD such as maladaptive guilt.

Children whose baseline narratives contained authoritative parenting themes had parents who displayed more positive parenting behavior and less negative parenting behavior during baseline observations coded by blind raters. On the other hand, children whose narratives contained authoritarian parenting themes had parents who displayed more negative parenting behavior and less positive parenting behavior during these observations. These results are consistent with previous findings by Donohue, et al. [15] that children’s negative or positive representations of their mothers measured via the MSSB correlated with negative and positive observed maternal behavior, respectively, and extend these findings by demonstrating for the first time that children’s narrative themes can validly reflect specific parenting styles. Given the importance of children’s representations of their caregivers in moderating the efficacy of PCIT-ED [15], and the role of authoritarian parenting as a risk factor for PO-MDD [24], capturing children’s valid internal representations of their caregivers’ parenting styles may allow us to more accurately predict risk for PO-MDD or identify specific dysfunctional aspects of the caregiver-child dyad to target in preventative interventions.

Though there were trends toward increased authoritative and decreased authoritarian parental representations following PCIT-ED, they were not significant. This finding was not entirely unexpected, given previous findings demonstrating a lack of change in observed parenting behaviors following PCIT-ED [45], despite changes in other parenting measures such as observed affect displays and positive interactional styles (overall warmth and a sense of connection between parent-child), as well as improved parental awareness of their own behaviors and affects. It is possible that at the follow-up assessment, insufficient time had passed for parenting behaviors to adjust to new parental awareness and interactional styles, and for these changes in parenting to become incorporated into the child’s internal working model of their caregiver’s parenting style; future studies should explore change in parenting themes at a more distal follow-up.

Our study had several limitations. First, some participants had to be excluded due to experimenter error, resulting in a relatively small sample size. Second, our data was collected in the context of long assessments lasting several hours; as a result, individual narrative tasks were kept relatively short, which may have limited children’s ability or willingness to express complex feelings or perseverate on their transgressions in ways that might reflect maladaptive reparative behaviors. It may be most effective to elicit complex guilt themes in the context of assessments focused exclusively on narratives which could provide more time for children to thoroughly express these internal feelings. Third, there was limited diversity in our study, with a predominantly white sample population. Findings should be replicated in a more diverse sample to examine whether they are generalizable. Fourth, although our study included families who fit well with MSSB-validated demographics, the MSSB was developed within the context of attachment theory, and therefore emphasizes mother-child relationships to the exclusion of other caregivers [9]. Future studies may adjust story stems further to capture diverse caregiving experiences and perspectives.

In sum, our study found that MSSB narratives validly capture preschool children’s perceptions of parenting styles, as well as expressions of guilt, with the latter decreasing following PCIT-ED, an evidence-based therapy for preschool depression. Our findings further validate the use of MSSB narratives as a useful source of preschool-age self-disclosure. Our findings suggest that narratives may be a valuable tool through which clinicians could monitor the course of specific depressive symptoms in young children. Furthermore, our findings support the efficacy of PCIT-ED as a treatment that ameliorates guilt in depressed preschoolers in addition to reducing depression symptoms generally.

## Supplementary Information

Below is the link to the electronic supplementary material.

ESM 1 (DOCX 35.6 KB)

## Data Availability

Data is provided within the manuscript or supplementary information file.
