# Supplementary material for: Evidence for decreased maladaptive guilt following PCIT-ED for depression as measured by story stem narratives: a promising method for preschool self-disclosure of emotions and experiences of parenting
Source: Eur Child Adolesc Psychiatry. 2026 Mar 18;35(7):2129–39. doi: 10.1007/s00787-026-02996-7 (PMC13427864; doi:10.1007/s00787-026-02996-7)
Supplement: Supplementary file 1 — (DOCX 35.6 KB) [file 787_2026_2996_MOESM1_ESM.docx]

**Online Resource 1**

*CONSORT Diagram of The Study*

Randomized into PCIT-ED Study (N=229)

Completed
Baseline Narratives
(N=226)

No Baseline Narratives (N=3)

No Post Narratives (N=49)

Completed
Post Narratives
(N=177)

Excluded Due to
Complete Disengagement
or Experimenter Error

Parenting Themes Codes
• Baseline only (N=14)
• Baseline and Post (N=9)
• Post only (N=26)

Guilt/Reparation Code
• Baseline only (N=17)
• Baseline and Post (N=11)
• Post only (N=27)

Included in Analyses

Parenting Themes Codes
• Baseline (N=154)
• Baseline and Post (N=128)

Guilt/Reparation Code
• Baseline (N=149)
• Baseline and Post (N=122)

**Online Resource 2**

*Descriptions of MSSB Narrative Story Stems Utilized in this Study*

**Baseline**

*Band-Aid*: the child is pretending to cook with a real knife, which is against the rules. The child cuts him or herself, and parents enter the room.

*Spilled Juice*: during a meal, the child reaches for and accidentally spills a container of juice set at the middle of the table.

**Post**

*Hot Soup*: impatient, the child reaches for and burns him or herself on the hot pot of soup that mother is cooking for dinner.

*Cookie Jar*: the child’s sibling steals a cookie and asks the child not to tell their parents. The parents enter the room.

**Online Resource 3**

*Descriptions of the guilt affect, adaptive reparation, and maladaptive reparation codes, along with cell sizes and analyses of the codes*

**Descriptions of supplemental codes**

*Guilt Affect*: this code reflects the extent to which a child expresses guilty emotional expression, including looking down in embarrassment. Guilt affect may be present when the child displays awareness of their fault in the transgression by apologizing. Guilt affect was coded as 0 when absent, 1 when present, and 2 when present to a particularly strong extent.

*Adaptive Reparation*: this code reflects the extent to which the child actively repairs the situation (e.g. apologizing for their mistake, caring for their own injury, or cleaning up their mess). Adaptive reparation was coded as 0 when absent, 1 when present.

*Maladaptive Reparation*: this code reflects the extent to which the child expresses negative affect while repairing, continues to repair after their transgression is resolved, is overly fixated on taking responsibility for repairing the situation following their transgression, or conducts the same reparative behavior repeatedly (e.g. apologizing multiple times). The child appears to be driven by excessive guilt. They may punish themselves. Maladaptive reparation was coded as 0 when absent and 1 when present.

**Cell sizes from all subjects at Baseline (N=154):**

*Given very few narratives were coded for 2 in Guilt Affect, those coded as 1 or 2 were collapsed into one cell.*

Guilt affect:

Absent: N=136

Present: N=18

Adaptive reparation:

Absent: N=124

Present: N=30

Maladaptive reparation:

Absent: N=150

Present: N=4

**Analytic Plan**

We analyzed whether guilt affect, adaptive reparation, or maladaptive reparation themes differed at Post as a function of randomization group (PCIT-ED vs Waitlist). Separate logistic regression models were conducted with each narrative guilt theme at Post as the outcome and randomization group as the independent variable. These models covaried for age, sex at birth, and the Baseline code corresponding to the outcome.

**Results**

            Online Resource 3.1 shows that guilt affect, adaptive reparation, and maladaptive reparation themes at Post did not significantly differ by randomization group.

**Online Resource 3.1**

*Logistic Regression Models of Guilt Affect, Adaptive Reparation, and Maladaptive Reparation in the Post Narratives by Randomization Group Covarying for Baseline Age, Sex at Birth, and Guilt Affect, Adaptive Reparation, or Maladaptive Reparation in the Baseline Guilt Narratives (N=128)*

| **DV = Post Guilt Affect** | **Est.** | **SE** | **OR (95% CI)** | **χ^2^** | **p** |
| --- | --- | --- | --- | --- | --- |
| Intercept | -4.638 | 3.140 | -- | 2.18 | 0.1396 |
| Baseline age | 0.338 | 0.552 | 1.40 (0.48, 4.14) | 0.37 | 0.5410 |
| Female sex | 0.084 | 0.500 | 1.18 (0.17, 8.39) | 0.03 | 0.8673 |
| Baseline guilt affect | 0.839 | 0.498 | 5.35 (0.76, 37.65) | 2.84 | 0.0918 |
| Waitlist vs. PCIT-ED | -0.325 | 0.503 | 0.52 (0.07, 3.74) | 0.42 | 0.5177 |
| **DV = Post Adaptive Reparation** | **Est.** | **SE** | **OR (95% CI)** | **χ^2^** | **p** |
| Intercept | -1.214 | 1.337 | -- | 0.82 | 0.3639 |
| Baseline age | -0.076 | 0.245 | 0.93 (0.57, 1.50) | 0.10 | 0.7564 |
| Female sex | -0.058 | 0.256 | 0.89 (0.33, 2.43) | 0.05 | 0.8225 |
| Baseline adaptive reparation | 0.058 | 0.310 | 1.12 (0.33, 3.79) | 0.04 | 0.8513 |
| Waitlist vs. PCIT-ED | -0.073 | 0.246 | 0.86 (0.33, 2.27) | 0.09 | 0.7675 |
| **DV = Post Maladaptive Reparation** | **Est.** | **SE** | **OR (95% CI)** | **χ^2^** | **p** |
| Intercept | -1.508 | 2.452 | -- | 0.38 | 0.5385 |
| Baseline age | -0.201 | 0.392 | 0.82 (0.38, 1.76) | 0.26 | 0.6088 |
| Female sex | -0.918 | 0.651 | 0.16 (0.01, 2.05) | 1.99 | 0.1586 |
| Baseline maladaptive reparation | 0.864 | 0.991 | 5.63 (0.12, 274.48) | 0.76 | 0.3832 |
| Waitlist vs. PCIT-ED | -0.061 | 0.407 | 0.89 (0.18, 4.36) | 0.02 | 0.8805 |

**Online Resource 4**

*Additional Descriptions of PCI Variables*

First, *Duration of Negative Parenting Behavior* was the mean duration of parent expressions of the following individual behavioral codes: Negative Physical Discipline, Negative Verbal Discipline, Intrusiveness, and Disengagement. Second, the *Duration of Negative Parenting Affect* was the mean duration of parent expressions of the following individual affective codes: Anger/Frustration and Sad/Anxious/Fearful/Worried. Third, *Duration of Positive Parenting Behavior* was the mean duration of parent expressions of the following individual codes: Physical Affection, Emotional Talk, Positive Reinforcement, Proactive Structure, Engagement, use of Rationale, and use of Directives. Finally, the *Duration of Positive Affect Composite* was the mean duration of parent expressions of the following individual codes: Low Intensity Positive Affect (e.g., closed mouth smiles; slightly positive warm tones) and High Intensity Positive Affect (e.g., open mouth smiles; regular positive fluctuations in voice tone such as excited yelling or high-pitched sounds; laughing; singing).

**Online Resource 5**

*Baseline Characteristics of Participants With vs. Without Post Narratives Data*

|  | **No Post Narratives**  **(N=49)** | | **Post**  **Narratives**  **(N=177)** | | **No Narratives**  **vs.**  **Narratives** | |
| --- | --- | --- | --- | --- | --- | --- |
| **Baseline Characteristic** | **%** | **n** | **%** | **n** | **χ^2^** | **p** |
| Female sex | 38.8 | 19 | 33.3 | 59 | 0.50 | 0.4782 |
| Hispanic ethnicity | 8.2 | 4 | 11.9 | 21 | 0.53 | 0.4648 |
| Race |  |  |  |  | F.E. | 0.0583 |
| White | 65.3 | 32 | 79.7 | 141 |  |  |
| Black | 22.5 | 11 | 9.0 | 16 |  |  |
| Asian | 0.0 | 0 | 0.6 | 1 |  |  |
| Multiracial | 12.2 | 6 | 10.7 | 19 |  |  |
|  | **Mean** | **SD** | **Mean** | **SD** | **t** | **p** |
| Age | 5.15 | 1.12 | 5.23 | 1.04 | -0.47 | 0.6392 |
| MDD severity score | 5.69 | 1.54 | 5.55 | 1.47 | 0.61 | 0.5444 |
| CBCL externalizing T-score | 66.82 | 9.71 | 66.42 | 10.21 | 0.24 | 0.8098 |
| CBCL internalizing T-score | 70.82 | 8.85 | 66.21 | 7.33 | 3.71 | 0.0003 |

F.E. = Fisher’s Exact Test

**Online Resource 6**

*IQ and Inattention in Participants With vs. Without Complete Disengagement in any Baseline or Post Narrative*

|  | **No Complete Disengagement**  **(N=159)** | | **Complete Disengagement**  **(N=16)** | | **No Disengagement**  **vs.**  **Disengagement** | |
| --- | --- | --- | --- | --- | --- | --- |
|  | **Mean** | **SD** | **Mean** | **SD** | **t** | **p** |
| KBIT-II IQ score | 107.60 | 13.58 | 96.73 | 16.36 | 2.91 | 0.0041 |
| CBCL inattention T-score | 60.04 | 8.10 | 60.81 | 11.66 | -0.26 | 0.7984 |
